# Growth differentiation factor 15 and early prognosis after out-of-hospital cardiac arrest

**DOI:** 10.1186/s13613-019-0593-9

**Published:** 2019-10-17

**Authors:** Ferran Rueda, Germán Cediel, Cosme García-García, Júlia Aranyó, Marta González-Lopera, M. Cruz Aranda Nevado, Judith Serra Gregori, Teresa Oliveras, Carlos Labata, Marc Ferrer, Nabil El Ouaddi, Antoni Bayés-Genís

**Affiliations:** 1 Heart Institute, Germans Trias i Pujol University Hospital, Carretera de Canyet s/n, Badalona, 08916 Barcelona, Spain; 2 Department of Medicine, CIBERCV, Autonomous University of Barcelona, Barcelona, Spain; 3 PhD Program in Internal Medicine, Autonomous University of Barcelona, Barcelona, Spain

**Keywords:** Out-of-hospital cardiac arrest, Prognostication, Neurologic outcome, Growth differentiation factor 15, Biomarkers

## Abstract

**Background:**

Growth differentiation factor 15 (GDF-15) is an inflammatory cytokine released in response to tissue injury. It has prognostic value in cardiovascular diseases and other acute and chronic conditions. Here, we explored the value of GDF-15 as an early predictor of neurologic outcome after an out-of-hospital cardiac arrest (OHCA).

**Methods:**

Prospective registry study of patients in coma after an OHCA, admitted in the intensive cardiac care unit from a single university center. Serum levels of GDF-15 were measured on admission. Neurologic status was evaluated according to the cerebral performance category (CPC) scale. The relationship between GDF-15 levels and poor neurologic outcome at 6 months was analyzed.

**Results:**

Among 62 patients included, 32 (51.6%) presented poor outcome (CPC 3–5). Patients with CPC 3–5 exhibited significantly higher GDF-15 levels (median, 17.1 [IQR, 11.1–20.4] ng/mL) compared to those with CPC 1–2 (7.6 [IQR, 4.1–13.1] ng/mL; *p* = 0.004). Multivariable logistic regression analyses showed that age (OR, 1.09; 95% CI 1.01–1.17; *p* = 0.020), home setting arrest (OR, 8.07; 95% CI 1.61–40.42; *p* = 0.011), no bystander cardiopulmonary resuscitation (OR, 7.91; 95% CI 1.84–34.01; *p* = 0.005), and GDF-15 levels (OR, 3.74; 95% CI 1.32–10.60; *p* = 0.013) were independent predictors of poor outcome. The addition of GDF-15 in a dichotomous manner (≥ 10.8 vs. < 10.8 ng/mL) to the resulting clinical model improved discrimination; it increased the area under the curve from 0.867 to 0.917, and the associated continuous net reclassification improvement was 0.90 (95% CI 0.48–1.44), which allowed reclassification of 37.1% of patients.

**Conclusions:**

After an OHCA, increased GDF-15 levels were an independent, early predictor of poor neurologic outcome. Furthermore, when added to the most common clinical factors, GDF-15 improved discrimination and allowed patient reclassification.

## Background

The prognosis remains poor for comatose survivors of an out-of-hospital cardiac arrest (OHCA) that are admitted to intensive care units, despite current aggressive care; survival rates are around 50%. Outcome is mostly related to the degree of hypoxic-ischemic brain injury, which leads to two-thirds of the mortality and persistent neurological disability in survivors [1–3]. Thus, early neurologic prognostication could be useful for avoiding futile treatments and providing information to relatives.

Unfortunately, among the available predictors of poor outcome, including clinical examination, electrophysiological tests, neuroinjury biomarkers, and neuroimaging, no single factor can predict poor outcome with certainty, particularly within the first 72 h after resuscitation. Moreover, some predictors can be affected by sedatives and therapeutic hypothermia. Therefore, accurate prognostication requires a multimodal approach that lasts several days in the majority of cases [4].

Although biomarkers represent a potential tool, they are still underused, especially because most current neuroinjury biomarkers lack consistent thresholds for identifying patients with no chance of survival. Moreover, further evidence is needed about the optimal time points for measuring them.

In the last decade, growth differentiation factor 15 (GDF-15), an inflammatory cytokine released in response to tissue injury, has emerged as a biomarker with prognostic value in cardiovascular diseases and other acute and chronic conditions. Moreover, GDF-15 provides information on the severity of disease [5]. In addition, we recently reported that admission levels of GDF-15 could serve as a robust, independent predictor of mortality in primary ventricular fibrillation, due to ST-segment elevation myocardial infarction (STEMI) [6], the most common cause of OHCA.

In view of this background, in the present study, we aimed to explore the value of GDF-15 on admission as early predictor of neurologic outcome in comatose survivors of OHCA.

## Methods

### Study population

We prospectively enrolled patients older than 18 years who were admitted in a persistent coma after successful resuscitation from an OHCA, to the intensive cardiac care unit from a single university center from April 2011 to May 2016. The diagnosis of cardiac arrest was established when patients exhibited an absence of spontaneous respiration, no palpable pulse, and no responsiveness to stimuli. Successful resuscitation was defined as recovery of blood pressure and pulse for more than 1 h, with or without continuous catecholamine infusion. Coma was defined as a Glasgow Coma Scale score < 9. Patients were excluded when the arrest arose from a non-cardiac origin (i.e., traumatic, toxic, or neurologic cause).

Pre-hospital data were recorded by emergency physicians, according to the Utstein style. Baseline demographics and clinical data were prospectively recorded. Treatment to restore spontaneous circulation and post-resuscitation care were provided according to international guidelines and recommendations [7, 8]. Patients were treated with hypothermia at 33 °C according to physician’s judgement and a local protocol, which excluded those with cardiopulmonary resuscitation (CPR) time longer than 45 min, non-shockable rhythms, refractory shock, refractory ventricular arrhythmias, severe coagulopathy or terminal disease.

The protocol was approved by the institutional Ethics Committee; all patients or their representatives provided written informed consent.

### Neurologic assessment and withdrawal of care

Sedation was discontinued at normothermia [24 h after return of spontaneous circulation (ROSC) in patients not treated with hypothermia]. In patients remaining comatose, a multimodal neurological evaluation was undertaken by a consultant neurologist a minimum of 72 h after normothermia (72 h after ROSC in patients not treated with hypothermia), according to current recommendations [4]. This evaluation was based on clinical findings together with electroencephalogram (EEG) and somatosensory evoked potentials (SSEP; only available from 2015). In addition, results of brain computed tomography (CT) and determinations of neuron-specific enolase (NSE; routinely measured from 2015), were considered. In patients treated with hypothermia, additional EEG was performed within the first 24 h. Brain death was diagnosed according to Spanish legislation. GDF-15 results were not available for the treating physicians and did not influence this process.

Full intensive care was provided until prognostic evaluation was completed. After a statement of “poor neurological prognosis”, decision on level of care was discussed with the patient’s family. Findings allowing withdrawal of life-sustaining therapies comprised: (1) brain death due to cerebral herniation; (2) persisting coma with a Glasgow motor scale (GMS) of 1–2, together with bilateral absence of N20-peak on SSEP, absence of pupillary and corneal reflexes, or presence of malignant EEG patterns (absence of reactivity, burst-suppression or refractory status epilepticus); (3) persisting coma (GMS 1–2) for more than 7 days after ROSC in absence of confounders, especially if elevated NSE levels (> 60 ng/mL at 48–72 h) or generalized ischemic changes on CT were present; (4) ethical reasons.

### GDF-15 samples

Blood samples were obtained on admission (baseline) and processed for central laboratory estimations of GDF-15. Serum was isolated by centrifugation and stored at − 80 °C until assayed. Patients without baseline levels of GDF-15 available were excluded from the study. Additional samples were drawn at other two pre-specified time points (12 h and 24 h after OHCA) for exploratory analyses.

### GDF-15 assay

Serum GDF-15 concentrations were determined with a fully automated electrochemiluminescence assay (ECLIA; Elecsys^®^ GDF-15 assay, Roche Diagnostics, Penzberg, Germany) on the Cobas Analytics e601 analyzer (Roche Diagnostics). The analytic performance of this assay had been validated, and it correlated closely with a previously established immunoradiometric assay method [9]. The Elecsys GDF-15 assay had a measuring range of 0.4 to 20 ng/mL, as stated by the manufacturer. Samples with values above the measuring range were diluted accordingly. The upper limit of the reference interval in healthy older individuals has been proposed to be 1.2 ng/mL.

### Clinical endpoint

The primary endpoint of the study was poor neurological outcome at 6 months, evaluated by the five-graded Cerebral Performance Category (CPC) scale [10]. A CPC score of 1 (good cerebral performance) or 2 (moderate cerebral disability) was considered a favorable outcome, and a CPC score of 3 (severe cerebral disability, conscious but dependent), 4 (coma) or 5 (death) was classified as a poor outcome. Follow-up was performed by the investigators, who were unaware of the GDF-15 results, with telephone interviews and by reviewing electronic patient records.

### Statistical analysis

Categorical variables are expressed as a number and percentage; continuous variables are expressed as the median and interquartile range (IQR). Comparisons of categorical variables were performed with the *χ*^2^ test. Comparisons of continuous variables were performed with the Wilcoxon’s rank sum test. Univariable and multivariable logistic regression models were performed with the backward stepwise procedure to determine whether baseline GDF-15 (logarithm transformed) constituted an independent predictor of poor neurological outcome. Variables that were not normally distributed were transformed to their natural logarithm. Odds ratios (ORs) with 95% confidence intervals (CIs) are reported. The following variables, obtained from the previously validated CAHP score [11], were incorporated into the regression model: age, delay between collapse and CPR, delay between CPR and ROSC, home setting arrest, no bystander CPR, non-shockable rhythm, dose of epinephrine, and pH at admission. Receiver operating characteristic (ROC) curve analyses were performed to evaluate whether baseline GDF-15 levels could predict poor neurological outcome at 6 months. We assessed whether there were any improvements in discrimination, calibration, and net reclassification by adding baseline GDF-15 to a clinical model, in a dichotomous manner, according to the best cutoff value derived from a ROC analyses. We performed ROC analyses and the Hosmer–Lemeshow test to obtain the net improvement in risk category reclassification (NRI). Differences were considered statistically significant at *p* < 0.05. All analyses were performed with STATA V.13.0 (StataCorp, College Station, TX).

## Results

### Study cohort characteristics

A total of 62 patients were included in the study. The flow chart depicting the population included is shown in Additional file 1: Figure S1. Comparison of baseline characteristics between included and excluded patients are detailed in Additional file 2: Table S1. The median age of participants was 59 (52–71) years, and 17.7% were women. Baseline characteristics of the study subjects, according to the presence or absence of the outcome, are detailed in Table 1.

**Table 1:** Baseline characteristics of the study cohort

Characteristics	All patients (*n* = 62)	Favorable outcome^a^ (*n* = 30)	Unfavorable outcome^a^ (*n* = 32)	*p* value
Demographics				
Age, years	59 (52–71)	56 (48–60)	67 (57–74)	0.002
Female sex	11 (17.7)	7 (23.3)	4 (12.5)	0.264
Clinical history				
Tobacco use	34 (54.8)	19 (63.3)	15 (46.9)	0.193
Arterial hypertension	34 (54.8)	12 (40.0)	22 (68.8)	0.023
Diabetes mellitus	15 (24.2)	2 (6.7)	13 (40.6)	0.002
Prior MI	10 (16.1)	3 (10.0)	7 (21.9)	0.204
CVD	6 (9.7)	2 (6.7)	4 (12.5)	0.438
Resuscitation variables				
Home setting arrest	23 (37.1)	5 (16.7)	18 (56.3)	0.001
Witnessed arrest	61 (98.4)	30 (100)	31 (96.9)	0.329
Bystander CPR	32 (51.6)	23 (76.7)	9 (28.1)	< 0.001
Collapse-CPR duration, min	4 (1–7)	3 (1–5)	5 (2–11)	0.004
CPR-ROSC duration, min	22 (13–30)	19 (11–31)	23 (15–30)	0.280
Non-shockable rhythm	10 (16.1)	1 (3.3)	9 (28.1)	0.008
Number of defibrillations	4 (2–6)	3 (2–6)	4 (1–6)	0.419
Epinephrine				0.004
0	12 (19.4)	11 (36.7)	1 (3.1)	
1–2 mg	19 (30.7)	7 (23.3)	12 (37.5)	
≥ 3 mg	31 (50.0)	12 (40.0)	19 (59.4)	
Admission GCS	3 (3–5)	5 (3–7)	3 (3–3)	< 0.001
Admission creatinine, (µmol/L)	114.9 (97.2–139.7)	109.6 (91.0–132.6)	128.6 (109.6–141.9)	0.033
Admission pH	7.22 (7.13–7.28)	7.25 (7.18–7.31)	7.19 (7.09–7.26)	0.065
Admission lactate, mmol/L^b^	4.4 (2.6–6.3)	3.4 (2.2–6.6)	5.2 (2.8–6.4)	0.337
Admission GDF-15, ng/mL	12.4 (5.7–19.6)	7.6 (41.4–13.0)	17.1 (11.1–20.4)	0.004
ICCU treatment				
Mechanical ventilation	62 (100)	30 (100)	32 (100)	–
Therapeutic hypothermia	37 (59.7)	20 (66.7)	17 (53.1)	0.277
Coronary angiography	52 (83.9)	28 (93.3)	24 (75.0)	0.050
Cardiac arrest etiology				
STEMI	38 (61.3)	20 (66.7)	18 (56.3)	0.400
NSTEMI	13 (21.0)	4 (13.3)	9 (28.1)	0.153
Vasospastic angina	4 (6.45)	3 (10.0)	1 (3.1)	0.271
Chronic CAD	3 (4.8)	0	3 (9.4)	0.086
Cardiomyopathy	2 (3.2)	2 (6.7)	0	0.138
Acute myocarditis	1 (1.6)	1 (3.3)	0	0.298
Others	1 (1.6)	0	1 (3.1)	0.329

Data are presented as the number of patients (%) or the median (IQR)

*MI* myocardial infarction, *CVD* cerebrovascular disease, *CPR* cardiopulmonary resuscitation, *ROSC* return of spontaneous circulation, *GCS* Glasgow Coma Scale, *ICCU* intensive cardiac care unit, *STEMI* ST-elevation myocardial infarction, *NSTEMI* non-ST-elevation myocardial infarction, *CAD* coronary artery disease

^a^Outcome favorability was based on the Cerebral Performance Category (CPC) score: scores 1–2 = favorable; scores 3–5 = unfavorable

^b^Estimation in 55 patients

In-hospital mortality was 50% (*n* = 31). The cause of death was brain injury in 83.9% of patients (*n* = 26; median survival 9 days [IQR, 6–11]), post-cardiac arrest shock and subsequent multiple organ failure in 12.9% (*n* = 4; median survival 3 days [IQR, 2.5–3]), and other in 3.2% (*n* = 1; survival 91 days). Among patients dying from neurologic injury, 11.5% (*n* = 3) were in cerebral death (median survival 3 days [IQR, 2.5–4.5]) and remaining 88.5% (*n* = 23) died after withdrawal of life-sustaining therapies (median survival 9 days [IQR, 7–11.5]). In those patients with poor outcome, neuroprognostication included two or more ancillary tests in 75% of them. Although EEG was the most widely used (81.2%), since SSEP were available, 81.8% of these patients were studied with them. Tests performed and their main results are detailed in Additional file 3: Table S2 and Additional file 4: Table S3.

**Fig. 1:**
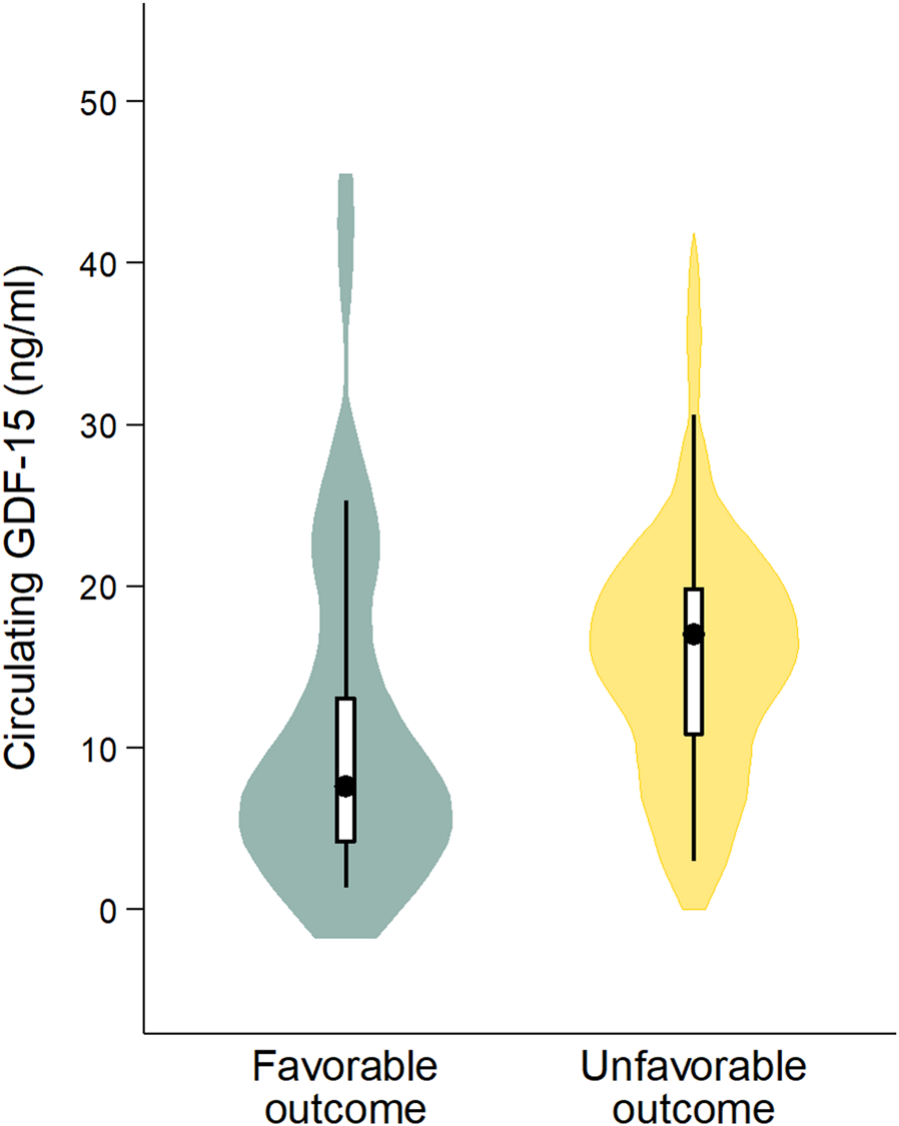
Violin plot showing the distribution of GDF-15 values in patients with and patients without adverse neurological outcome

Poor neurological outcome at 6 months occurred in 51.6% (*n* = 32) of patients. Baseline GDF-15 levels were significantly higher in patients with CPC 3–5 compared to those with CPC 1–2 (median, 17.1 [IQR, 11.1–20.4] ng/mL vs. 7.6 [IQR, 4.1–13.1] ng/mL; *p* = 0.004) (Fig. 1). In the 34 patients in which this data was available, the blood samples corresponding to baseline levels were obtained a median of 138 [IQR, 115–200] min after arrest.

### Predictors of poor neurological outcome

**Fig. 2:**
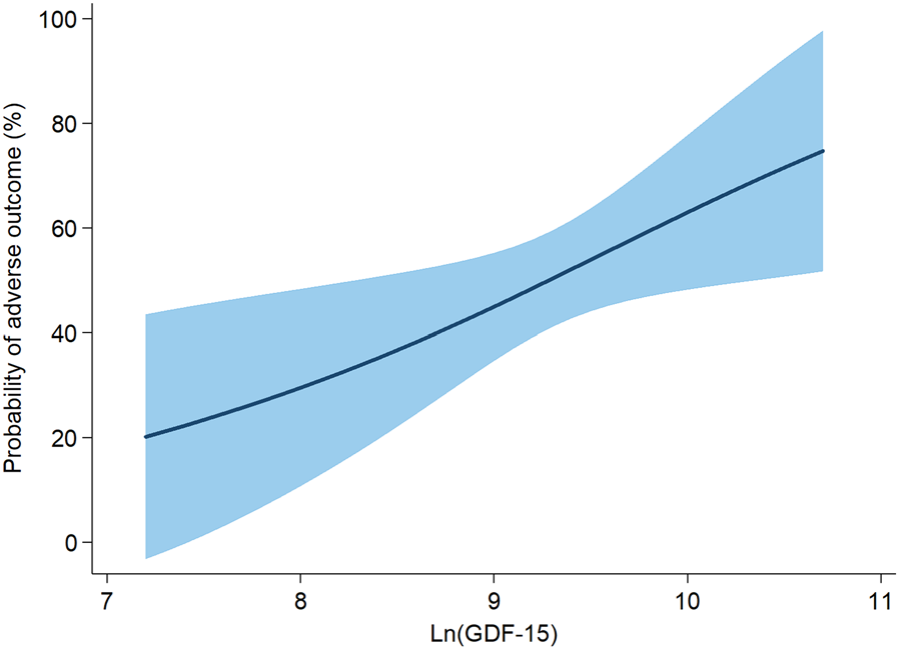
Graph plot showing predictive margins (solid line), with 95% confidence intervals (shaded area), of adverse neurological outcome, according to GDF-15 levels. GDF-15 was transformed on a natural logarithmic scale

Multivariable logistic regression analyses showed that age (OR, 1.09; 95% CI 1.01–1.17; *p* = 0.020), home setting arrest (OR, 8.07; 95% CI 1.61–40.42; *p* = 0.011), no bystander CPR (OR, 7.91; 95% CI 1.84–34.01; *p* = 0.005), and baseline GDF-15 levels (OR, 3.74; 95% CI 1.32–10.60; *p* = 0.013) were independent predictors of the occurrence of the primary endpoint (CPC 3–5) (Table 2). Moreover, the predictive margins of adverse neurological outcomes were higher in individuals with high baseline GDF-15 levels, compared to those with low baseline GDF-15 levels (Fig. 2).

**Table 2:** Results of univariable and multivariable logistic regression analyses for identifying predictors of neurological outcome at 6 months

	Univariable logistic regression			Multivariable logistic regression		
	OR	95% CI	*p*	OR	95% CI	*p*
Age, years	1.07	1.02–1.13	0.004	1.09	1.01–1.17	0.020
Collapse-CPR duration^a^	2.17	1.24–3.79	0.006			
CPR-ROSC duration^a^	1.74	0.65–4.68	0.269			
Home setting arrest	6.43	1.96–21.07	0.002	8.07	1.61–40.42	0.011
No bystander CPR	8.40	2.67–26.37	< 0.001	7.91	1.84–34.01	0.005
Non-shockable rhythm	11.35	1.34–96.18	0.026			
Epinephrine, mg						
0	1					
1–2	18.86	1.99–178.8	0.010			
≥ 3	17.42	1.99–152.7	0.010			
Admission creatinine	4.87	1.14–20.88	0.033			
Admission pH	0.034	0.001–1.754	0.093			
Admission lactate^b^	1.54	0.65–3.64	0.321			
Admission GDF-15^a^	2.88	1.40–5.92	0.004	3.74	1.32–10.60	0.013

Multivariate results are presented after backward elimination was completed

*CPR* cardiopulmonary resuscitation, *ROSC* return of spontaneous circulation

^a^Transformed on a natural logarithmic scale

^b^Estimation in 55 patients

In an exploratory analysis, we studied the dynamics of GDF-15 within the first 24 h after admission in the 58 patients in which these samples were available. We found a descending pattern, with a higher median GDF-15 concentration at baseline than at 12 or at 24 h. This pattern was observed in patients with and without adverse neurological outcomes. However, at all 3 specified time points, GDF-15 levels were higher in patients with poor neurological outcomes than in patients with favorable neurological outcomes. This pattern was also observed in patients with OHCA secondary to STEMI and in patients treated with therapeutic hypothermia (Additional file 5: Figure S2 and Additional file 6: Figure S3). Additionally, in another exploratory analysis, we analyzed associations between GDF-15 levels at 12 h and 24 h (one model for each specified time-point) and the clinical endpoint. We found associations that remained significant, even after a multivariable logistic regression analysis (OR, 3.90; CI 95% 1.15–13.26; *p* = 0.029 and OR, 3.65; CI 95%, 1.42–9.44; *p* = 0.007, respectively) (Additional file 7: Table S4, Additional file 8: Table S5). Considering that levels of GDF-15 at 12 h and 24 h could have been influenced in TTM group, baseline characteristics of patients according to TTM treatment are detailed in Additional file 9: Table S6.

### Incremental prognostic value of GDF-15 over clinical risk factors

**Fig. 3:**
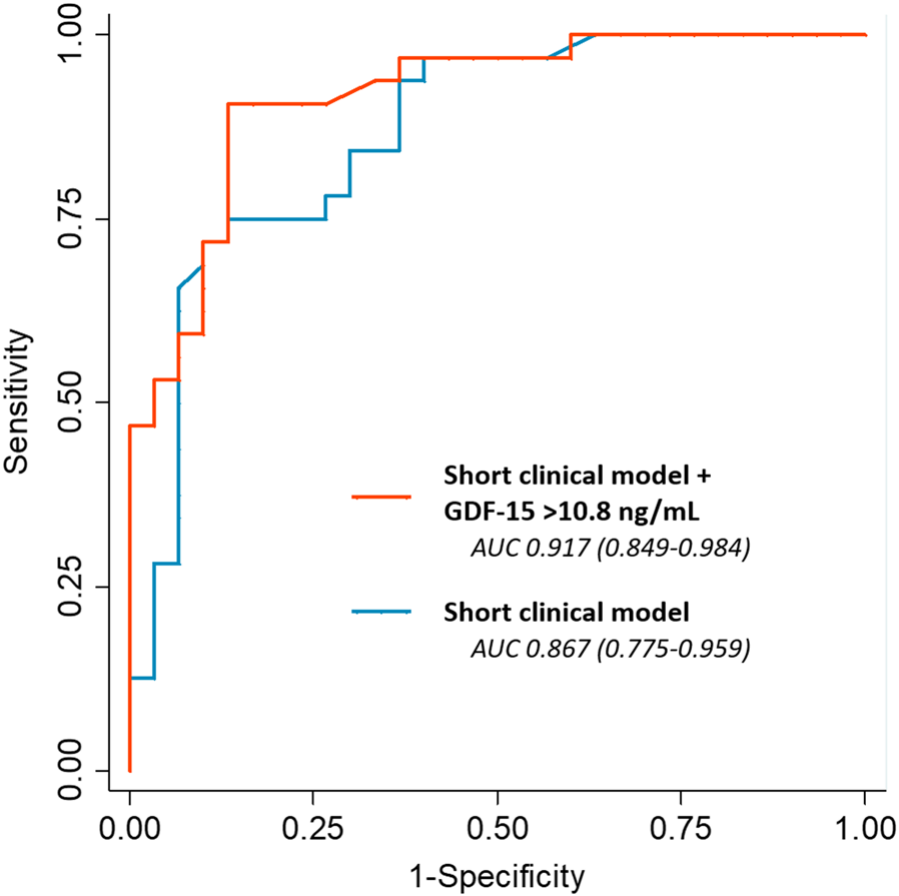
ROC curves showing the accuracy of GDF-15 for predicting poor neurologic outcome at 6 months, when added in a dichotomous manner (≥ 10.8 ng/mL) to the short clinical model

We found that 10.8 ng/ml was the optimal GDF-15 cutoff level for maximum classification efficiency (Table 3). First, we evaluated the addition of GDF-15, in a dichotomous manner (≥ 10.8 ng/mL vs. < 10.8 ng/mL), to a short clinical model, which contained variables significantly associated with adverse outcome after a multivariable regression analysis (i.e., age, home setting arrest, and no bystander CPR). We found that the addition of GDF-15 improved discrimination. The area under the curve (AUC) increased from 0.867 (CI 95% 0.775–0.959) to 0.917 (CI 95% 0.849–0.984) (Fig. 3), and the associated continuous NRI was 0.90 (CI 95% 0.48–1.44), which allowed the reclassification of 37.1% (CI 95% 11.3–54.8) of patients. Similarly, the addition of GDF-15 to an extended clinical model (i.e., the short clinical model, plus the collapse-to-CPR duration, non-shockable rhythm, and epinephrine) improved discrimination. The AUC increased from 0.895 (CI 95% 0.818–0.972) to 0.942 (CI 95% 0.886–0.997), and the associated continuous NRI was 1.15 (CI 95% 0.32–1.73), which allowed the reclassification of 32.3% (CI 95% 4.8–46.8) of patients. Additional file 10: Table S7 summarizes the calibration, discrimination, and reclassification metrics used.

**Table 3:** Sensitivity and specificity for the prediction of poor outcome for different baseline GDF-15 cutoff values

Cutoff (ng/mL)	Sensitivity (%)	Specificity (%)	Correctly classified (%)
1.6	100	3.3	53.2
4.5	90.6	30.0	61.3
7.0	84.4	46.7	66.1
*10.8* ^a^	*78.1*	*66.7*	*72.6*
12.5	68.8	70.0	69.3
15.0	62.5	76.7	69.4
17.5	46.9	76.7	61.3
20.9	25.0	83.3	53.2
27.1	15.6	96.7	54.8

^a^Optimal cutoff point for maximum efficiency (false negative cost = false positive cost)

## Discussion

In this study, we found that the circulating GDF-15 level, measured at admission after an OHCA, was an independent and early predictor of poor neurologic outcome at 6 months. In addition, when the GDF-15 level was added to a clinical model that included the usual clinical predictors available shortly after admission, it improved discrimination, and one-third of patients could be reclassified. GDF-15 levels showed a large, early increase after an OHCA, followed by a sustained decline during the first 24 h.

Prognostication after an OHCA remains challenging, particularly regarding brain damage, and accurate evaluation usually needs several days [4, 8]. Unlike other predictors, like examination or EEG, biomarkers can provide early, quantitative data, independent of the effects of sedatives. Consequently, they represent a growing area of interest. Several markers of brain damage have been studied [12], in particular NSE, which is the only included in current clinical guidelines [4, 8]. However, the present role of biomarkers is limited to cases in which more robust predictors, such as physical examination or SSEP, provide inconclusive results. Despite providing a notable discrimination, their main limitation is the difficulty in establishing a consistent threshold with a zero false positive rate. In addition, another weakness of most proposed neuroinjury biomarkers is a delayed release to blood in response to ischemia/reperfusion processes. The peaks of blood levels, and as a result the optimal time point for measuring, are usually observed from 24 h after ROSC. In the case of NSE, blood levels peak at 48–96 h after an arrest [13] and, accordingly, the highest prognostic accuracy has been reported at 48 h post-ROSC, or when measured serially during the first 72 h [14]. Protein S-100 exhibits an earlier peak and could provide a predictive value similar to NSE already at 24 h from the arrest [15, 16]. Secretoneurin [17] and glial fibrillary acidic protein [18] have also been evaluated, but they showed a lower discrimination and no temporal advantage respect NSE and S-100. In recent years, two new biomarkers, tau protein and neurofilament light chain (NFL), have been proposed. Serum tau may offer better diagnostic accuracy than NSE for poor outcome, although the highest predictive values are likewise observed at 48–72 h from ROSC [19]. By contrast, NFL seems superior to other biomarkers (NSE, S-100 and tau) when assessed as early as 24 h after cardiac arrest [20]. Nevertheless, despite these promising results, more data should be available before its routine use in prognostication.

GDF-15 is a stress-responsive member of the transforming growth factor-β (TGF-β) cytokine superfamily. GDF-15 is weakly expressed in tissues, including the central nervous system [21], under normal conditions. Although its pathobiology is not fully understood, it is strongly induced by macrophages in response to inflammation and tissue injury. Thus, circulating levels of GDF-15 have been identified as an inflammatory biomarker with prognostic value in several conditions, particularly in cardiovascular diseases. In the acute setting, increased levels are a robust predictor of organ dysfunction and death from acute myocardial infarction [6, 22, 23] to cardiogenic shock [24]. GDF-15 also serves as biomarker in other critical disease conditions, such as acute pulmonary embolism [25], acute respiratory distress syndrome [26], or sepsis [27]. On the other hand, at lower cut-off values, GDF-15 levels can predict long-term cardiovascular events, bleeding, cancer, and all-cause mortality, both in patients with chronic heart diseases and in individuals that dwell in community settings [5]. Thus, GDF-15 also represents a marker of biological age and chronic disease burden.

In the present study, blood GDF-15 increased rapidly to high levels after circulation was restored; this increase was followed by a decline during the first 24 h (Additional file 5: Figure S2). Neuronal damage can be a source of circulating GDF-15. In support of this hypothesis, experimental data have demonstrated that GDF-15 was locally overexpressed in rodent models of brain injury [28, 29]. Furthermore, other studies have described an association between rising GDF-15 levels and functional outcome after an ischemic stroke [30, 31]. Interestingly, a small study noted a significant correlation between the levels of GDF-15 and S-100, as well as similar discriminative capabilities [30]. However, the great releasing of GDF-15 after OHCA seems to respond mainly to the very early global inflammatory response related to post-cardiac arrest syndrome (PCAS), rather than ongoing brain injury.

PCAS is a unique, complex combination of pathological processes, which include brain injury, myocardial dysfunction, systemic ischemia/reperfusion responses, and often, the unresolved disease process that caused the cardiac arrest [32]. Severity of PCAS is a major determinant of outcome [33, 34], largely dependent on the duration of the whole body ischemia and reperfusion injury, which are also the main trigger of the inflammatory system. Therefore, there is a close association between the severity of PCAS, the magnitude of the inflammatory response, the severity of organ dysfunction and neurologic outcome, as inferred from small previous studies on inflammatory biomarkers in this clinical setting. Copeptin levels on admission have been associated with death and subsequent organ failure in one study [35], and were predictive for neurological outcome in another [36], when measured within 48 h after ROSC. Circulating procalcitonin were related with the severity of PCAS and predicted neurological outcome accurately already at 12 h from the arrest [33]. More recently, interleukin-6 emerged as a potential early biomarker. Its levels on admission, but not the high-sensitivity C-reactive protein or the S-100 ones, have been associated with extra-cerebral organ dysfunction and were independent predictors for poor neurologic outcome [37]. Nevertheless, it is uncertain whether IL-6 can provide incremental value above traditional factors associated with a poor prognosis [38].

It is clear that GDF-15 shares with the previously discussed neuroinjury biomarkers the same limitations regarding the possibility of false positives. In addition, GDF-15 is not specific for neuronal damage. This can explain why we observed lower AUCs for poor outcome prediction than the reported ones from neuroinjury biomarkers (usually ≥ 0.90) when measured at their optimal time points. Even so, we should understand that biomarkers are a means to explore the different pathways involved in PCAS. They offer complementary and sequential information. Inflammatory biomarkers, such as GDF-15, could provide additional value to neuroinjury markers, particularly in the early acute phase [39]. Thus, to maximize the predictive value of biomarkers, instead of employing a single, best biomarker strategy, a multimarker approach may be more advantageous [12]. This may include acute-phase, myocardial dysfunction and neuroinjury biomarkers, summarizing the main components of PCAS. Several scores based on clinical parameters rapidly available on admission have been developed and validated [11, 40]. Further research might address whether the addition of information from biomarkers could provide enough accuracy to guide decision-making and information to relatives in the first hours after arrest.

## Limitations

Several limitations of our study should be noted. First and foremost, it was performed at a single center and involved a small number of patients, which limited the generalization of our observations. Included population was limited to OHCA of cardiac cause, and external validity is restricted to these patients. Second, other biomarkers were not analyzed. NSE was only available in 27 (43.5%) patients. Analyses lacked statistical power to extract conclusions about its relationship with GDF-15. However, the study was focused on variables with established predictive value short after admission, and NSE is useful a minimum of 24 h after arrest. Third, in many cases, clinical outcome was determined by the withdrawal of life support. Thus, the outcome could have been affected by a so-called “self-fulfilling prophecy”. Nevertheless, treating physicians were not aware of GDF-15 measurements, since analyses were performed after treatment of all patients was completed. More studies are needed to validate our results externally and evaluate the clinical utility of GDF-15 in this group of patients.

## Conclusions

In comatose patients that survived an OHCA, high circulating GDF-15 levels on admission were an independent and early predictor of severe neurologic disability at 6 months. When added to the most common clinical factors associated with a poor outcome, GDF-15 improved discrimination and allowed patient reclassification. Further studies are needed to assess whether the incorporation of GDF-15 to a multimodal risk stratification approach might provide early and accurate neurologic prognostication.

## Supplementary information

**Additional file 1: Figure S1.** Flow chart of patients included in the study.

**Additional file 2: Table S1.** Baseline characteristics of patients included in the study in comparison with those excluded because measurement of GDF-15 on admission or other relevant data were missing.

**Additional file 3: Table S2.** Distribution and results of clinical and ancillary examinations in all patients included in the study.

**Additional file 4: Table S3.** Distribution and results of clinical and ancillary examinations in patients who died in-hospital from neurological causes.

**Additional file 5: Figure S2.** GDF-15 levels during the first 24 h of admission in patients with and without adverse neurological outcomes. Comparisons between groups based on the Mann–Whitney test.

**Additional file 6: Figure S3.** GDF-15 levels during the first 24 h of admission, according to therapeutic hypothermia treatment (TTM) and ST-elevation myocardial infarction (STEMI).

**Additional file 7: Table S4.** Results from univariable and multivariable logistic regression analyses, including GDF-15 levels measured at 12 h.

**Additional file 8: Table S5.** Results from univariable and multivariable logistic regression analyses, including GDF-15 levels measured at 24 h.

**Additional file 9: Table S6.** Baseline characteristics of patients who received targeted temperature management in comparison with those who did not.

**Additional file 10: Table S7.** Performance of models for the neurological outcome at 6 months.

## Data Availability

The datasets used and/or analysed during the current study are available from the corresponding author on reasonable request.

## Abbreviations

OHCA: out-of-hospital cardiac arrest
GDF-15: growth differentiation factor 15
STEMI: ST-segment elevation myocardial infarction
CPR: cardiopulmonary resuscitation
ROSC: return of spontaneous circulation
EEG: electroencephalogram
SSEP: somatosensory evoked potentials
CT: computed tomography
NSE: neuron-specific enolase
GMS: Glasgow motor scale
CPC: cerebral performance category
IQR: interquartile range
OR: odds ratio
CI: confidence interval
ROC: receiver operating characteristic
NRI: net reclassification improvement
AUC: area under the curve
NFL: neurofilament light chain
PCAS: post-cardiac arrest syndrome
