# Supplementary figures and images for: Growth differentiation factor 15 and early prognosis after out-of-hospital cardiac arrest
Source: Ann Intensive Care. 2019 Oct 17;9:119. doi: 10.1186/s13613-019-0593-9 (PMC6797678; doi:10.1186/s13613-019-0593-9)

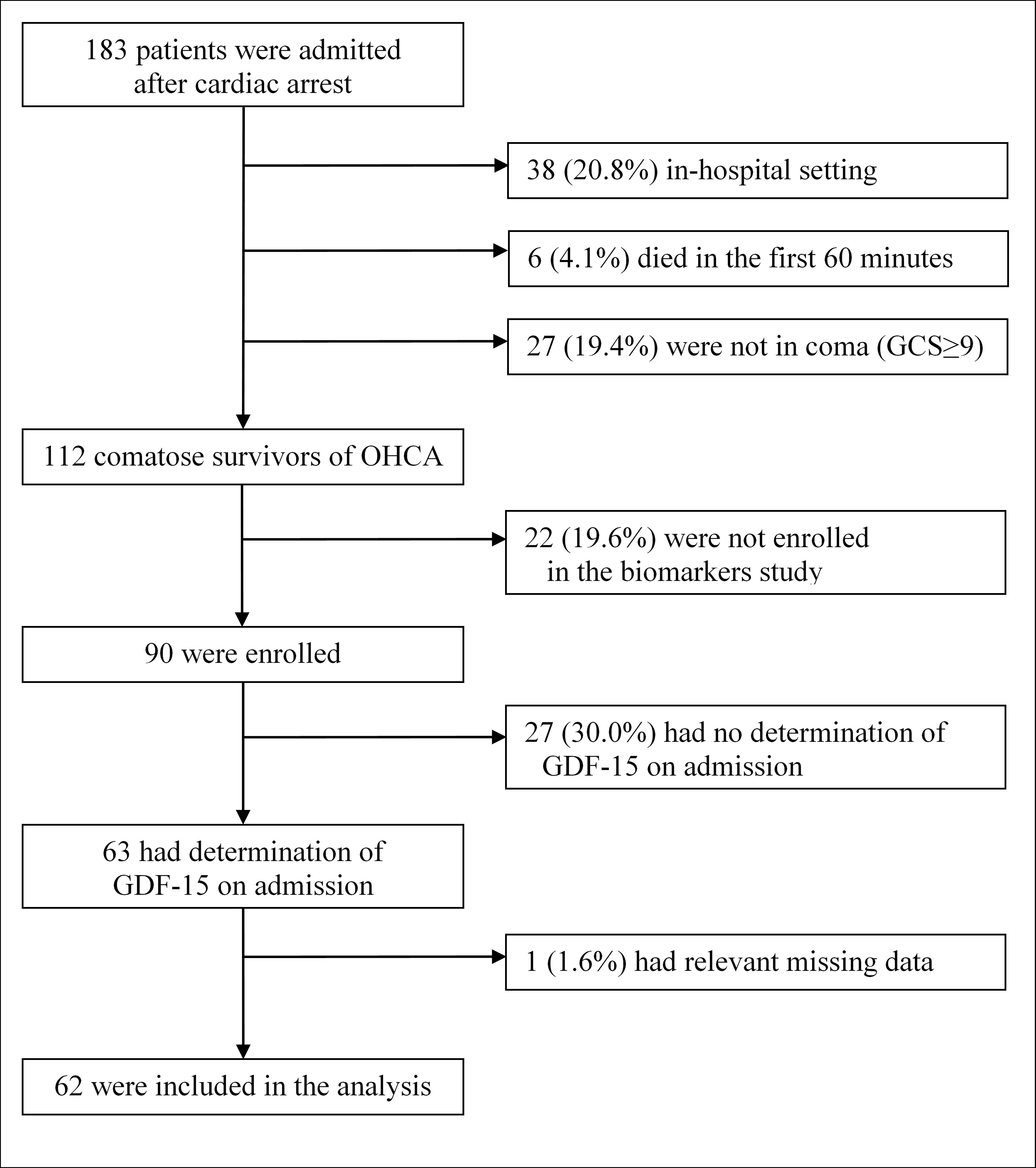

Supplement: Supplementary file 1 — Additional file 1: Figure S1. Flow chart of patients included in the study. [file 13613_2019_593_MOESM1_ESM.tif]

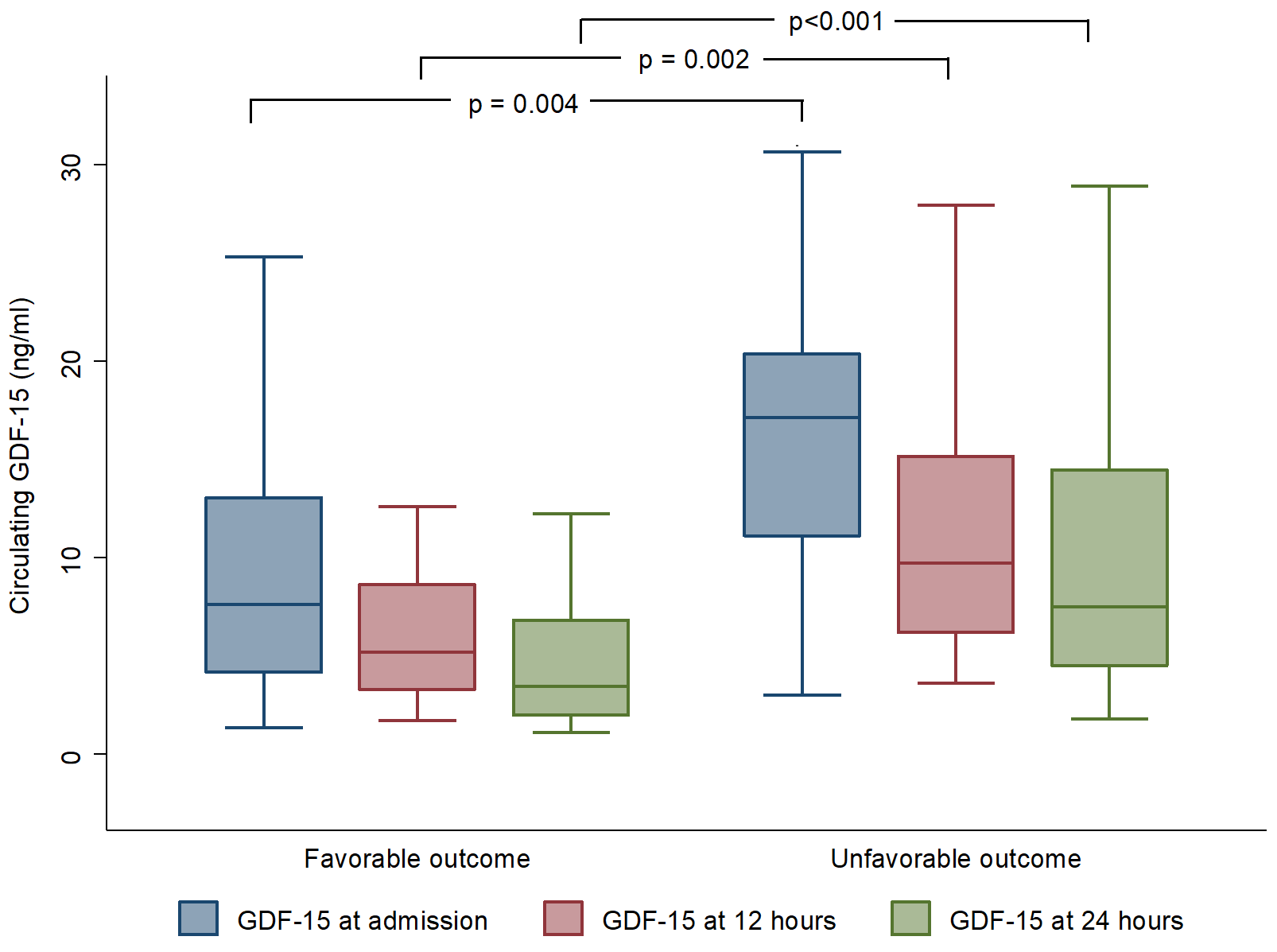

Supplement: Supplementary file 5 — Additional file 5: Figure S2. GDF-15 levels during the first 24 h of admission in patients with and without adverse neurological outcomes. Comparisons between groups based on the Mann–Whitney test. [file 13613_2019_593_MOESM5_ESM.tif]

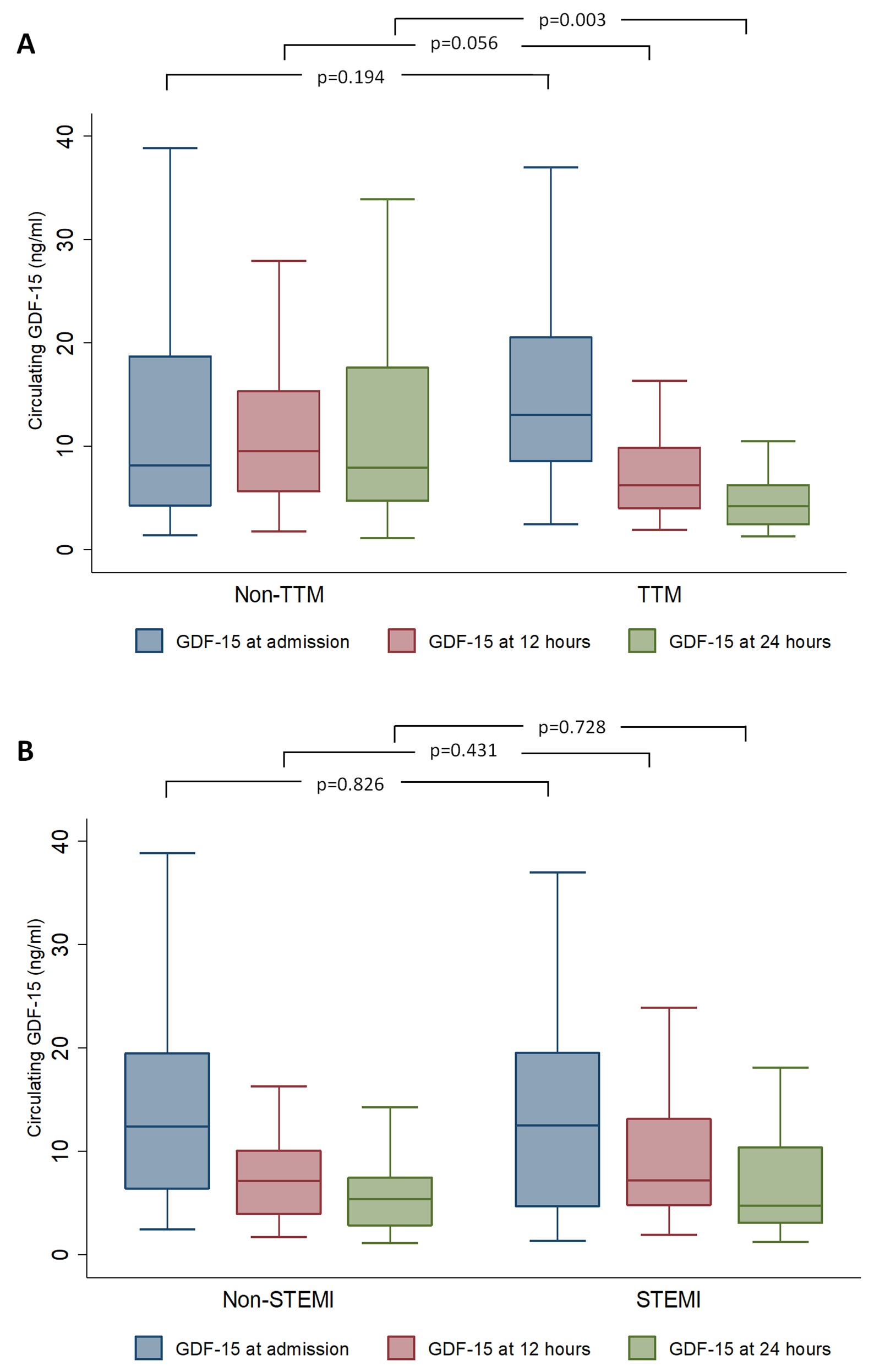

Supplement: Supplementary file 6 — Additional file 6: Figure S3. GDF-15 levels during the first 24 h of admission, according to therapeutic hypothermia treatment (TTM) and ST-elevation myocardial infarction (STEMI). [file 13613_2019_593_MOESM6_ESM.tif]
