# Supplementary material for: Growth differentiation factor 15 and early prognosis after out-of-hospital cardiac arrest
Source: Ann Intensive Care. 2019 Oct 17;9:119. doi: 10.1186/s13613-019-0593-9 (PMC6797678; doi:10.1186/s13613-019-0593-9)
Supplement: Supplementary file 2 — Additional file 2: Table S1. Baseline characteristics of patients included in the study in comparison with those excluded because measurement of GDF-15 on admission or other relevant data were missing. [file 13613_2019_593_MOESM2_ESM.docx]

**Additional Table S1.** Baseline characteristics of patients included in the study in comparison with those excluded because measurement of GDF-15 on admission or other relevant data were missing.

| **Characteristics** | **All patients (n = 90)** | **Included (n = 62)** | **Not included (n = 28)** | **P value** |
| --- | --- | --- | --- | --- |
| ***Demographics*** |  |  |  |  |
| Age, years | 59 (53-69) | 59 (52-71) | 59 (53-68) | 0.931 |
| Female sex | 18 (20.0) | 11 (17.7) | 7 (25.0) | 0.425 |
| ***Clinical History*** |  |  |  |  |
| Tobacco use | 43 (47.8) | 34 (54.8) | 9 (32.1) | 0.046 |
| Arterial hypertension | 50 (55.6) | 34 (54.8) | 16 (57.1) | 0.839 |
| Diabetes mellitus | 23 (25.6) | 15 (24.2) | 8 (28.6) | 0.659 |
| Prior MI | 19 (21.1) | 10 (16.1) | 9 (32.1) | 0.085 |
| CVD | 8 (8.9) | 6 (9.7) | 2 (7.1) | 0.696 |
|  |  |  |  |  |
| ***Resuscitation variables*** |  |  |  |  |
| Home setting arrest | 40 (44.4) | 23 (37.1) | 17 (60.7) | 0.037 |
| Witnessed arrest | 84 (93.3) | 61 (98.4) | 23 (82.1) | 0.004 |
| Bystander CPR | 42 (46.7) | 32 (51.6) | 10 (35.7) | 0.162 |
| Collapse-CPR duration, min | 5 (2-9) | 4 (1-7) | 6 (4-10) | 0.027 |
| CPR-ROSC duration, min | 22 (13-31) | 22 (13-30) | 22 (14-35) | 0.727 |
| Collapse-ROSC duration, min | 26 (20-38) | 26 (19-37) | 29 (24-42) | 0.206 |
| Non-shockable rhythm | 16 (17.8) | 10 (16.1) | 6 (21.4) | 0.543 |
| Number of defibrillations | 3 (2-5) | 4 (2-6) | 2 (1-5) | 0.140 |
| Epinephrine |  |  |  | 0.320 |
| 0 | 14 (15.6) | 12 (19.4) | 2 (7.1) |  |
| 1-2 mg | 28 (31.1) | 19 (30.7) | 9 (32.1) |  |
| ≥ 3 mg | 48 (53.3) | 31 (50.0) | 17 (60.7) |  |
| Admission GCS | 3 (3-5) | 3 (3-5) | 3 (3-3) | 0.004 |
| Admission creatinine, (µmol/L) | 127.8 (101.7-141.5) | 114.9 (97.2-139.7) | 132.6 (122.9-154.7) | 0.033 |
| Admission pH* | 7.22 (7.13-7.30) | 7.22 (7.13-7.28) | 7.25 (7.15-7.31) | 0.367 |
|  |  |  |  |  |
| ***ICCU treatment*** |  |  |  |  |
| Mechanical ventilation | 90 (100) | 62 (100) | 28 (100) | - |
| Therapeutic hypothermia | 55 (61.1) | 37 (59.7) | 18 (64.3) | 0.678 |
| Coronary angiography | 71 (78.9) | 52 (83.9) | 19 (67.9) | 0.085 |
|  |  |  |  |  |
| ***Cardiac arrest etiology*** |  |  |  | <0.001 |
| STEMI | 41 (45.6) | 38 (61.3) | 3 (10.7) |  |
| NSTEMI | 17 (18.9) | 13 (21.0) | 4 (14.3) |  |
| Vasospastic angina | 4 (4.4) | 4 (6.45) | 0 |  |
| Chronic CAD | 15 (16.7) | 3 (4.8) | 12 (42.9) |  |
| Cardiomyopathy | 4 (4.4) | 2 (3.2) | 2 (7.1) |  |
| Acute myocarditis | 1 (1.1) | 1 (1.6) | 0 |  |
| Others | 2 (2.2) | 1 (1.6) | 1 (3.6) |  |

Data are presented as the number of patients (%) or the median (IQR). MI, myocardial infarction; CVD, cerebrovascular disease; CPR, cardiopulmonary resuscitation; ROSC, return of spontaneous circulation; GCS, Glasgow Coma Scale; ICCU, Intensive Cardiac Care Unit; STEMI, ST-Elevation Myocardial Infarction; NSTEMI, Non-ST-Elevation Myocardial Infarction; CAD, Coronary Artery Disease; CPC, Cerebral Performance Category score. *Estimation in 88 patients (not available in 2 patients from the not included group).
