# Supplementary material for: Growth differentiation factor 15 and early prognosis after out-of-hospital cardiac arrest
Source: Ann Intensive Care. 2019 Oct 17;9:119. doi: 10.1186/s13613-019-0593-9 (PMC6797678; doi:10.1186/s13613-019-0593-9)
Supplement: Supplementary file 3 — Additional file 3: Table S2. Distribution and results of clinical and ancillary examinations in all patients included in the study. [file 13613_2019_593_MOESM3_ESM.docx]

**Supplemental Table 2.** Distribution and results of clinical and ancillary examinations in all patients included in the study.

|  | **All patients**  **(n = 62)** | **Favorable outcome^a^ (n = 30)** | **Unfavorable outcome^a^ (n = 32)** |
| --- | --- | --- | --- |
| **Physical examination** | **62 (100)** | **30 (100)** | **32 (100)** |
| No pupillary and corneal reflexes | 9 (14.5) | 0 | 9 (28.1) |
|  |  |  |  |
| **EEG** | **34 (54.8)** | **8 (26.7)** | **26 (81.2)** |
| Malignant patterns^b^ | 15 (44.1) | 0 | 15 (57.7) |
| Benign patterns | 19 (55.9) | 8 (100) | 11 (42.3) |
|  |  |  |  |
| **SSEP** | **12 (19.4)** | **3 (10.0)** | **9 (28.1)** |
| N20 absent | 5 (41.7) | 0 | 5 (55.6) |
| Other patterns | 37 (58.3) | 3 (100) | 4 (44.4) |
|  |  |  |  |
| **Computed tomography** | **32 (51.6)** | **10 (33.3)** | **22 (68.8)** |
| Global edema / Decreased grey/white matter differentiation | 11 (34.4) | 0 | 11 (50.0) |
| Other findings | 21 (65.6) | 10 (100) | 11 (50.0) |
|  |  |  |  |
| **Neuron-specific enolase** | **27 (43.5)** | **11 (36.7)** | **16 (50.0)** |
| >60 mcg/L at 48-72h | 7 (26.9) | 0 | 7 (43.8) |
| <60 mcg/L at 48-72h | 20 (74.1) | 11 (100) | 9 (56.2) |
|  |  |  |  |
| **At least 1 test** | **46 (74.2)** | **16 (53.3)** | **30 (93.8)** |
| **At least 2 tests** | **35 (56.5)** | **11 (36.7)** | **24 (75.0)** |
| **At least 3 tests** | **16 (25.8)** | **3 (10.0)** | **13 (40.6)** |
| **All 4 tests** | **8 (12.9)** | **2 (6.7)** | **6 (18.8)** |

Data are presented as the number of patients (%). ^a^Outcome favorability was based on the Cerebral Performance Category (CPC) score: scores 1-2 = favorable; scores 3-5 = unfavorable. ^b^Malignat EEG patterns include: absence of EEG reactivity to external stimuli, presence of burst-suppression or status epilepticus at ≥72 h after ROSC. EEG, electroencephalogram; SSEP, somatosensory evoked potentials.
