# Supplementary material for: Growth differentiation factor 15 and early prognosis after out-of-hospital cardiac arrest
Source: Ann Intensive Care. 2019 Oct 17;9:119. doi: 10.1186/s13613-019-0593-9 (PMC6797678; doi:10.1186/s13613-019-0593-9)
Supplement: Supplementary file 4 — Additional file 4: Table S3. Distribution and results of clinical and ancillary examinations in patients who died in-hospital from neurological causes. [file 13613_2019_593_MOESM4_ESM.docx]

**Supplemental Table 3.** Distribution and results of clinical and ancillary examinations in patients who died in-hospital from neurological causes.

|  | **All neurological deaths (n = 26)** | **Cerebral death (n = 3)** | **WLST**  **(n = 23)** |
| --- | --- | --- | --- |
| **Physical examination** | **26 (100)** | **3 (100)** | **23 (100)** |
| No pupillary and corneal reflexes | 8 (30.8) | 3 (100) | 5 (21.7) |
|  |  |  |  |
| **EEG** | **24 (92.3)** | **2 (66.7)** | **22 (95.7)** |
| Malignant patterns^b^ | 15 (62.5) | 2 (100) | 13 (59.1) |
| Benign patterns | 9 (37.5) | 0 | 9 (40.9) |
|  |  |  |  |
| **SSEP** | **9 (34.6)** | **1 (33.3)** | **8 (34.8)** |
| N20 absent | 5 (55.6) | 1 (100) | 4 (50.0) |
| Other patterns | 4 (44.4) | 0 | 4 (50.0) |
|  |  |  |  |
| **Computed tomography** | **19 (73.1)** | **3 (100)** | **16 (69.6)** |
| Global edema / Decreased grey/white matter differentiation | 11 (57.9) | 2 (66.7) | 9 (56.2) |
| Other findings | 8 (42.1) | 1 (33.3) | 7 (43.8) |
|  |  |  |  |
| **Neuron-specific enolase** | **14 (53.8)** | **1 (33.3)** | **13 (56.5)** |
| >60 mcg/L at 48-72h | 6 (42.9) | 0 | 6 (46.2) |
| <60 mcg/L at 48-72h | 8 (57.1) | 1 (100) | 7 (53.8) |
|  |  |  |  |
| **At least 1 test** | **26 (100)** | **3 (100)** | **23 (100)** |
| **At least 2 tests** | **22 (84.6)** | **3 (100)** | **19 (82.6)** |
| **At least 3 tests** | **12 (46.2)** | **1 (33.3)** | **11 (47.8)** |
| **All 4 tests** | **6 (23.1)** | **0** | **6 (26.1)** |

Data are presented as the number of patients (%). ^a^Malignat EEG patterns include: absence of EEG reactivity to external stimuli, presence of burst-suppression or status epilepticus at ≥72 h after ROSC. WLST, withdrawal of life-sustaining therapies. EEG, electroencephalogram; SSEP, somatosensory evoked potentials.
