# Supplementary material for: Growth differentiation factor 15 and early prognosis after out-of-hospital cardiac arrest
Source: Ann Intensive Care. 2019 Oct 17;9:119. doi: 10.1186/s13613-019-0593-9 (PMC6797678; doi:10.1186/s13613-019-0593-9)
Supplement: Supplementary file 7 — Additional file 7: Table S4. Results from univariable and multivariable logistic regression analyses, including GDF-15 levels measured at 12 h. [file 13613_2019_593_MOESM7_ESM.docx]

**Additional Table S4.** Results from univariable and multivariable logistic regression analyses, including GDF-15 levels measured at 12 h.

|  | **Univariable**  **logistic regression** | | | **Multivariable**  **logistic regression** | | |
| --- | --- | --- | --- | --- | --- | --- |
|  | **OR** | **95% CI** | **p** | **OR** | **95% CI** | **p** |
| Age, years | 1.07 | 1.02-1.13 | 0.004 | 1.09 | 1.02-1.17 | 0.013 |
| Collapse-to-CPR duration* | 2.17 | 1.24-3.79 | 0.006 |  |  |  |
| CPR-to-ROSC duration* | 1.74 | 0.65-4.68 | 0.269 |  |  |  |
| Home setting arrest | 6.43 | 1.96-21.07 | 0.002 | 5.33 | 1.02-27.73 | 0.047 |
| No bystander CPR | 8.40 | 2.67-26.37 | <0.001 | 7.60 | 1.67-34.53 | 0.009 |
| Non-shockable rhythm | 11.35 | 1.34-96.18 | 0.026 |  |  |  |
| Epinephrine, mg |  |  |  |  |  |  |
| 0 | 1 |  |  |  |  |  |
| 1-2 | 18.86 | 1.99-178.8 | 0.010 |  |  |  |
| ≥ 3 | 17.42 | 1.99-152.7 | 0.010 |  |  |  |
| Admission creatinine | 4.87 | 1.14-20.88 | 0.033 |  |  |  |
| Admission pH | 0.034 | 0.001-1.754 | 0.093 |  |  |  |
| GDF-15 at 12-h*† | 3.60 | 1.47-8.80 | 0.005 | 3.90 | 1.15-13.26 | 0.029 |

CPR, cardiopulmonary resuscitation; ROSC, return of spontaneous circulation. Multivariate results are presented after backward elimination was completed. *Transformed on a natural logarithmic scale. †N=58.
