# Supplementary material for: Growth differentiation factor 15 and early prognosis after out-of-hospital cardiac arrest
Source: Ann Intensive Care. 2019 Oct 17;9:119. doi: 10.1186/s13613-019-0593-9 (PMC6797678; doi:10.1186/s13613-019-0593-9)
Supplement: Supplementary file 9 — Additional file 9: Table S6. Baseline characteristics of patients who received targeted temperature management in comparison with those who did not. [file 13613_2019_593_MOESM9_ESM.docx]

**Additional Table S6.** Baseline characteristics of patients who received targeted temperature management in comparison with those who did not.

| **Characteristics** | **All patients**  **(n = 62)** | **No TTM group**  **(n = 25)** | **TTM group**  **(n = 37)** | **P value** |
| --- | --- | --- | --- | --- |
| ***Demographics*** |  |  |  |  |
| Age, years | 59 (52-71) | 67 (56-73) | 58 (50-64) | 0.038 |
| Female sex | 11 (17.7) | 6 (24.0) | 5 (13.5) | 0.289 |
| ***Clinical History*** |  |  |  |  |
| Tobacco use | 34 (54.8) | 13 (52.0) | 21 (56.8) | 0.712 |
| Arterial hypertension | 34 (54.8) | 12 (48.0) | 22 (59.5) | 0.374 |
| Diabetes mellitus | 15 (24.2) | 8 (32.0) | 7 (18.9) | 0.238 |
| Prior MI | 10 (16.1) | 7 (28.0) | 3 (8.1) | 0.037 |
| CVD | 6 (9.7) | 3 (12.0) | 3 (8.1) | 0.611 |
|  |  |  |  |  |
| ***Resuscitation variables*** |  |  |  |  |
| Home setting arrest | 23 (37.1) | 13 (52.0) | 10 (27.0) | 0.046 |
| Witnessed arrest | 61 (98.4) | 24 (96.0) | 37 (100) | 0.220 |
| Bystander CPR | 32 (51.6) | 12 (48.0) | 20 (54.1) | 0.640 |
| Collapse-CPR duration, min | 4 (1-7) | 5 (1-11) | 4 (1-5) | 0.514 |
| CPR-ROSC duration, min | 22 (13-30) | 20 (13-25) | 22 (13-30) | 0.495 |
| Non-shockable rhythm | 10 (16.1) | 10 (40.0) | 0 | <0.001 |
| Number of defibrillations | 4 (2-6) | 2 (1-5) | 4 (3-6) | 0.075 |
| Epinephrine |  |  |  | 0.967 |
| 0 | 12 (19.4) | 5 (20.0) | 7 (18.9) |  |
| 1-2 mg | 19 (30.7) | 8 (32.0) | 11 (29.7) |  |
| ≥ 3 mg | 31 (50.0) | 12 (48.0) | 19 (51.4) |  |
| Admission GCS | 3 (3-5) | 4 (3-5) | 3 (3-5) | 0.224 |
| Admission creatinine, (µmol/L) | 114.9 (97.2-139.7) | 128.2 (111.4-139.7) | 112.3 (91.1-132.6) | 0.123 |
| Admission pH | 7.22 (7.13-7.28) | 7.22 (7.13-7.31) | 7.20 (7.15-7.27) | 0.802 |
| Admission lactate, mmol/L* | 4.4 (2.6-6.3) | 6.3 (5-9) | 3.2 (2.15-5.5) | <0.001 |
| Admission GDF-15, ng/mL | 12.4 (5.7-19.6) | 8.2 (4.2-18.7) | 13.0 (8.5-20.5) | 0.194 |
|  |  |  |  |  |
| ***ICCU treatment*** |  |  |  |  |
| Coronary angiography | 52 (83.9) | 22 (88.0) | 30 (81.1) | 0.467 |
|  |  |  |  |  |
| ***Cardiac arrest etiology*** |  |  |  |  |
| STEMI | 38 (61.3) | 15 (60.0) | 23 (62.2) | 0.864 |
| NSTEMI | 13 (21.0) | 6 (24.0) | 7 (18.9) | 0.630 |
| Vasospastic angina | 4 (6.45) | 0 | 4 (10.8) | 0.141 |
| Chronic CAD | 3 (4.8) | 1 (4.0) | 2 (5.4) | 1.000 |
| Cardiomyopathy | 2 (3.2) | 1 (4.0) | 1 (2.7) | 1.000 |
| Acute myocarditis | 1 (1.6) | 1 (4.0) | 0 | 0.403 |
| Others | 1 (1.6) | 1 (4.0) | 0 | 0.403 |

Data are presented as the number of patients (%) or the median (IQR *Estimation in 55 patients. TTM, targeted temperature management; MI, myocardial infarction; CVD, cerebrovascular disease; CPR, cardiopulmonary resuscitation; ROSC, return of spontaneous circulation; GCS, Glasgow Coma Scale; ICCU, Intensive Cardiac Care Unit; STEMI, ST-Elevation Myocardial Infarction; NSTEMI, Non-ST-Elevation Myocardial Infarction; CAD, Coronary Artery Disease.
