# Supplementary material for: Growth differentiation factor 15 and early prognosis after out-of-hospital cardiac arrest
Source: Ann Intensive Care. 2019 Oct 17;9:119. doi: 10.1186/s13613-019-0593-9 (PMC6797678; doi:10.1186/s13613-019-0593-9)
Supplement: Supplementary file 10 — Additional file 10: Table S7. Performance of models for the neurological outcome at 6 months. [file 13613_2019_593_MOESM10_ESM.docx]

**Additional Table S7.** Performance of models for the neurological outcome at 6 months.

| Predictive model | **C-statistic (95% CI)** | **HL (p value)** | **NRI (95% CI)** | **Reclassification % (95% CI)** |
| --- | --- | --- | --- | --- |
| GDF-15 >10.8 ng/mL | 0.724 (0.611-0.836) | 10.14 (0.25) |  |  |
| Short clinical model^a^ | 0.867 (0.775-0.959) | 13.4 (0.10) |  |  |
| Short clinical model + GDF-15 >10.8 ng/mL | 0.917 (0.849-0.984) | 5.97 (0.65) | 0.90 (0.48-1.44) | 37.1 (11.3-54.8) |
| Extended clinical model^b^ | 0.895 (0.818-0.972) | 6.77 (0.56) |  |  |
| Extended clinical model + GDF-15 >10.8 ng/mL | 0.942 (0.886-0.997) | 5.89 (0.66) | 1.15 (0.32-1.73) | 32.3 (4.8-46.8) |
|  |  |  |  |  |

^a^Short clinical model was generated based on variables significantly associated with adverse outcome after a multivariable logistic regression analysis (i.e., age, home setting arrest, and no bystander CPR).

^b^Extended clinical model was generated based on variables associated with adverse outcome after a univariable logistic regression analysis (i.e., age, home setting arrest, no bystander CPR, collapse-to-CPR duration, non-shockable rhythm, and epinephrine).

CI, confidence interval; HL, Hosmer Lemeshow; GDF, growth differentiation factor.
